# Advancing Brain Tumor Classification through Fine-Tuned Vision Transformers: A Comparative Study of Pre-Trained Models

**DOI:** 10.3390/s23187913

**Published:** 2023-09-15

**Authors:** Abdullah A. Asiri, Ahmad Shaf, Tariq Ali, Muhammad Ahmad Pasha, Muhammad Aamir, Muhammad Irfan, Saeed Alqahtani, Ahmad Joman Alghamdi, Ali H. Alghamdi, Abdullah Fahad A. Alshamrani, Magbool Alelyani, Sultan Alamri

**Affiliations:** 1 Radiological Sciences Department, College of Applied Medical Sciences, Najran University, Najran 61441, Saudi Arabia; 2 Department of Computer Science, COMSATS University Islamabad Sahiwal Campus, Sahiwal 57000, Pakistan; 3 Electrical Engineering Department, College of Engineering, Najran University, Najran 61441, Saudi Arabia; 4 Radiological Sciences Department, College of Applied Medical Sciences, Taif University, Taif 21944, Saudi Arabia; 5 Department of Radiological Sciences, Faculty of Applied Medical Sciences, The University of Tabuk, Tabuk 47512, Saudi Arabia; 6 Department of Diagnostic Radiology Technology, College of Applied Medical Sciences, Taibah University, Madinah 41477, Saudi Arabia; 7 Department of Radiological Sciences, College of Applied Medical Science, King Khalid University, Abha 61421, Saudi Arabia

**Keywords:** brain tumor classification, pre-trained ViT, fine-tuning, medical image analysis, cutting-edge models

## Abstract

This paper presents a comprehensive study on the classification of brain tumor images using five pre-trained vision transformer (ViT) models, namely R50-ViT-l16, ViT-l16, ViT-l32, ViT-b16, and ViT-b32, employing a fine-tuning approach. The objective of this study is to advance the state-of-the-art in brain tumor classification by harnessing the power of these advanced models. The dataset utilized for experimentation consists of a total of 4855 images in the training set and 857 images in the testing set, encompassing four distinct tumor classes. The performance evaluation of each model is conducted through an extensive analysis encompassing precision, recall, F1-score, accuracy, and confusion matrix metrics. Among the models assessed, ViT-b32 demonstrates exceptional performance, achieving a high accuracy of 98.24% in accurately classifying brain tumor images. Notably, the obtained results outperform existing methodologies, showcasing the efficacy of the proposed approach. The contributions of this research extend beyond conventional methods, as it not only employs cutting-edge ViT models but also surpasses the performance of existing approaches for brain tumor image classification. This study not only demonstrates the potential of ViT models in medical image analysis but also provides a benchmark for future research in the field of brain tumor classification.

## 1. Introduction

A brain tumor refers to the unregulated and irregular growth of cells in the brain and the adjacent tissues. These growths come in two types: benign (non-cancerous) and malignant (cancerous). They may develop within the brain tissue or originate in other areas of the body and metastasize to the brain [1]. Benign and malignant development can come from spreading cells or tissues in the surrounding tissues or from the brain cells. As per the data provided by the Central Brain Tumor Registry of the United States (CBTRUS), statistics indicate that more than 700,000 individuals are impacted by an initial-stage brain tumor. The signs of a brain tumor include persistent headaches, seizures, visual changes, balance problems, cognitive changes, and personality shifts. Symptoms can vary based on tumor type, location, and size [2]. The membrane tissues of the brain give rise to meningiomas, which are non-cancerous brain tumors. The pituitary gland, a significant brain organ, is the location of another type of tumor. Considering these unique features of brain tumors, doctors can correctly identify and treat brain tumors [3]. With the advent of deep learning techniques, fine-tuned pre-trained convolutional neural network (CNN) models [4] and ViTs [5] have emerged as powerful tools in the field of medical image analysis, enabling accurate and efficient brain tumor detection.

CNNs are widely used for image analysis tasks because they capture intricate features hierarchically. Fine-tuning involves taking a pre-trained CNN model on a large dataset and adapting it to a specific task, such as brain tumor detection. The pre-trained CNN models are initially trained on massive image datasets like ImageNet, learning general features. Fine-tuning involves removing the last few layers and adding new ones trained on the target dataset to adapt to the new task [6]. ViTs have gained popularity as an alternative to CNNs for image analysis tasks. They break down images into fixed-size patches and process them with self-attention mechanisms, capturing global relationships among pixels. Input images are divided into fixed-size patches, which are linearly embedded before being fed into the transformer architecture [7]. MRI can find brain tumors using magnets and radio waves [8], and X-rays are used in this method to find brain tumors.

Specimen collection is the most reliable surgical procedure for identifying brain tumors. The kind of brain tumor is identified by microscopically examining a tiny part of the tumor. These imaging methods work well to find brain tumors [9]. In addition, the tumor’s dimensions, position, and other factors can affect how accurate these imaging techniques are in adjacent tissue. Furthermore, the size, position, and presence of adjacent tissues may determine how accurate these imaging techniques are. The detection of brain tumors also makes use of a number of machine learning and deep learning techniques to overcome the problems mentioned above. Based on the provided image, the model predicts brain tumors’ existence or absence [10]. Brain tumors may be accurately identified by an algorithm based on supervised machine learning (SVM) through classification and segmentation modeling. Brain tumor diagnosis also makes use of the deep learning method CNN or conventional neural network. This program recognizes brain tumors by extracting features from brain images and classifying them by those features [11]. In [12], the author used an already trained (CNN) model as a base and updated it to recognize the various forms of brain tumors. This model is known as the VGG-16 model. A demonstration of 3064 MRI scans and images can be found in the dataset from 233 patients, initially utilized for learning the model and then checking the output produced by the algorithm.

In order to determine whether variations in data gathering and processing practices between institutions would impact the algorithm’s performance, they evaluated it using data from two more groups. According to the findings, CNN performed more effectively when learned on images or scans from the same group or institution than when evaluated on information obtained from a different group or institution. The BraTS-Net, a deep CNN framework that the authors suggest, combines modules (the most popular are inception and residual) and 2D and 3D transformations to enhance performance. The research team employed the BraTS 2013 dataset, encompassing MRI images and diagnostic data pertaining to individuals afflicted by edema, necrosis, and low-grade gliomas (LGGs). In reference to [13], the authors designed a CNN architecture characterized by three convolutional layers and two fully connected layers. This architecture was trained using a compilation of MRI images derived from patients whose tumor diagnoses employed the BAT technique. To conduct their study, the authors harnessed the BraTS 2015 dataset, containing MRI imaging records for patients with Glioma. These BraTS datasets, comprising images and scans from patients afflicted with gliomas, serve as training and evaluation resources for researchers’ algorithms. The researchers’ findings indicated that their model outperformed U-Net, Deep Medic, and BraTS benchmark techniques, exhibiting sensitivity scores of 0.783, a typical DSC score of 0.814, and a specificity score of 0.999.

Using MRI scans and pictures, the authors undertake tumor identification and classification using YOLOv2, NSGA, LDA, SVM, and the k-NN inception version 3 [14]. They used BraTS, a dataset that includes MRI scans from cancer patients together with HGG and LGG, to train their algorithm. It is still being determined how the model will operate on multiple datasets or with different kinds of brain tumors because the authors only test it on one dataset. A unique method for accurately identifying and segmenting tumors from MRI imaging data was put forth in [15]. The suggested architecture of the bitr-unit model comprises two important parts: a decoder (based on transforms) and an encoder (based on CNNs). The authors used the publicly accessible dataset BraTS2021, which allowed them to assess the results and functionality of their model. According to the data, the Bitr-unit model, which was the working model, performed better in separation, with a total DSC score for the entire tumor of 0.908. The Swin Transformer’s architecture, which the authors modified to accommodate medical image segmentation tasks, has proven to be an effective framework for natural image categorization issues. The authors used this BraTS 2021 dataset to see how well the model performed in accurate segmentation while being quicker and more memory efficient. The authors in the paper propose the volumetric transformer network (VTN), a unique architecture that extends the conventional transformers structure to deal with 3D volumetric data [16].

The Headset of the Head and Neck Auto-segmentation Task (Hecktor) dataset and the authors used the Brain Tumor Segments 2021 dataset to test this technique. The authors claimed that VTN performs better than other ML methods while being faster and more memory-effective regarding segmentation accuracy. In an earlier investigation [17], scholars employed a transformation network to address the complexities inherent in multimodal brain tumor differentiation classification issues (utilizing MRI), encompassing challenges such as tumor tissue variability and diversity. This network has the capacity to capture intricacies spanning multiple modalities. The researchers juxtaposed their methodology against various machine learning approaches from the BRATS 2019–2020 dataset. The findings showcased a dice similarity coefficient (DSC) score of 90% for the entire tumor in the context of BRATS 2019–2020. The fact that the researchers thoroughly tested their methodology on two sizable datasets made available to the public is one of the study’s strengths because it increased the accuracy and generalizability of the findings. To use the technology as a clinically approved technique, the computational requirements for retraining and reasoning must be disclosed by the authors as this could be a major hurdle.

Artificial intelligence and deep learning algorithms can identify brain tumors but have some limitations. These algorithms’ output depends on the data’s accuracy and volume. These algorithms might not be able to distinguish between complex kinds of tumors that are uncommon and unusual brain tumors’ precision can also be affected by features selection, model design, and other parameters [18]. Using a neural network framework, the ViT technique offers a novel approach to computer vision problems [19]. ViT is a distinctive technique for computer vision applications due to its neural network design. By breaking up the input data into portions of images and using self-attention approaches, the ViTs model can detect complex cancers.

During testing, a probability map that provides details on the areas of the brain tumors is produced as a result [20]. ViT models may accurately diagnose brain tumors using MRI and image data. Brain tumor images are automatically processed using ViT to extract features, which are then used to identify tumors. There are several brain tumor datasets available, one notable collection being the BraTS dataset. This dataset includes magnetic resonance imaging (MRI) scans and images, and focuses on the most common and most dangerous type of brain tumor.

This work uses fine-tuned convolutional neural network (CNN) and ViT models to detect and classify brain tumors. There are five ViT models, R50-ViT-l16, ViT-b16, ViT-l16, ViT-l32, and ViT-b32, used for detecting and classifying brain tumors. Each model has a different architecture and number of parameters. The contributions of the work are:

- ViT models can be effectively used for brain tumor image classification, even without any task-specific training.
- Fine-tuning of ViT models can significantly improve their performance on brain tumor images.
- A comprehensive evaluation of ViT models for brain tumor image classification, comparing them to existing approaches.
- The proposed models have the potential to improve the accuracy and efficiency of brain tumor image classification, which could lead to earlier diagnosis and treatment for patients.

The structure of the following paper comprises a methodology section, which explains the overall methodology workflow with different parameters of models. The results section explains the results of all the models with their evaluation schemes, and the last one is the conclusion of the paper, which concludes all the work performed during the research work.

## 2. Materials and Methods Section

### 2.1. Dataset

**Figure 1:**
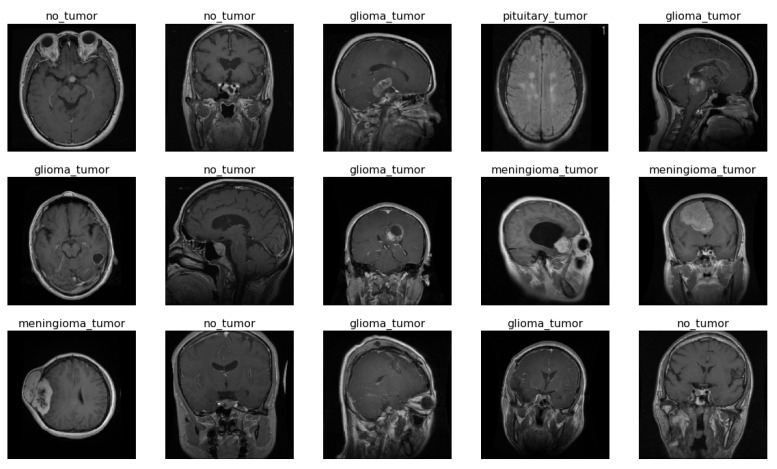
Sample images of dataset.

A collection of 5712 MRI images of human brains, categorized with one of four classes as shown in Figure 1, make up the brain tumor MRI dataset which is available on Kaggle. Glioma: glial cells, which nourish and protect neurons, are the origin of a particular type of brain tumor. Meningioma: A tumor that develops in the membranes that envelop the brain and spinal cord, or meninges. No tumor: A negative result on a brain scan for a tumor. Pituitary: The pituitary gland, a little organ near the base of the brain, can develop tumors. As stated in Table 1, the dataset is separated into two folders—training: The 4855 MRI scans in this folder were used to train the suggested model; and testing: The 857 MRI scans in this folder were used to test various model assumptions.

### 2.2. Data Pre-Processing and Augmentation

In our study, we performed the following data preprocessing steps: **Data Collection and Labeling:** We collected a diverse set of brain tumor images from the Kaggle dataset. These images were organized into categories corresponding to different tumor types, including glioma, meningioma, pituitary tumors, and a “no tumor” class. **Data Splitting:** We divided the dataset into training, validation, and testing sets using the ‘train_test_split’ function from Scikit-Learn. This allowed us to assess the model’s performance on different data subsets and ensure robustness. **Image Resizing:** To ensure uniformity in input dimensions, we resized all images to a fixed size of 224 × 224 pixels using the target_size parameter in the Keras ImageDataGenerator. **Color Mode:** We maintained the RGB color mode for all images to work with the standard format commonly used in deep learning tasks. **Label Encoding:** We encoded the categorical labels into numerical format using one-hot encoding. This transformation facilitated the use of categorical cross-entropy loss during model training. **Data Augmentation:** To enhance the model’s ability to generalize and improve its robustness, we applied data augmentation techniques during training. These techniques were integrated into the data pipeline using the Keras ImageDataGenerator. Augmentation techniques included random rotations, flips, zoom, and brightness adjustments. Data augmentation artificially increases the diversity of the training data, helping the model learn more effectively.

### 2.3. Vision Transformer Architecture

The vision transformer (ViT) architecture is a cutting-edge method of locating brain malignancies. This technique divides the input image into smaller patches, each subjected to an independent neural network analysis. When the results from these patches are combined, the network can understand the overall features and structure of the complete image. The ViT model can effectively handle the challenging task of brain tumor diagnosis in MRI scans because of this patch-wise analysis and self-attention mechanisms.

ViT models do not require sequential input data. Unlike recurrent neural networks (RNNs) or transformers for natural language processing (NLP), ViT models process images as a whole without relying on the sequential order. Each image is treated as a set of patches, and the model extracts features from these patches in parallel.

#### 2.3.1. Key Components of ViT Architecture

The patch embedding projection and the class token projection are two essential elements of the ViT architecture. These two vital linear transformations play a central role in the architecture. The patch embedding projection serves to convert individual flattened patch vectors into lower-dimensional vectors complete with their distinctive features. Through this approach, the model becomes adept at capturing significant features from specific patches. In contrast, the class token projection operates by transforming a trainable class token vector into a feature vector with reduced dimensions. This class token serves the purpose of amalgamating information from all patches and thereby represents the entirety of the image.

The multihead self-attention (MSA) module undertakes the transformation of input vectors into query, key, and value vectors, a pivotal step preceding the computation of the ultimate output. This procedure empowers the model to apprehend intricate interdependencies and interactions among diverse constituents of the image. Employing scaled dot-product attention, the MSA module computes the weighted summation of values by considering the attention weights associated with each value. To ensure the stability and efficacy of training within deep neural networks, techniques such as layer normalization and residual connections are applied to the resultant output.

An integral element within the ViT architecture is the feedforward neural network (FFN) module. Comprising a duo of linear layers, each is succeeded by a rectified linear unit (ReLU) activation function, and this module is essential. These successive layers effectuate the conversion of the output from the MSA module into a multitude of feature vectors. Each of these vectors corresponds to a specific patch within the input image and delineates essential characteristics of said patch. Furthermore, the FFN module incorporates several instances of layer normalization. These additional normalization stages play a pivotal role in reinforcing the stability of the training process and enhancing the overall efficacy of the ViT model.

#### 2.3.2. Pre-Trained ViT Models

Each model has a different architecture and number of parameters, as shown in Table 1, and all models are defined in the model description section. The five specific ViT models that were used in this study were:

1. R50-ViT-L16
2. ViT-b16
3. ViT-l16
4. ViT-l32
5. ViT-b32

#### 2.3.3. Fine-Tuning of Pre-Trained Models

**Figure 2:**
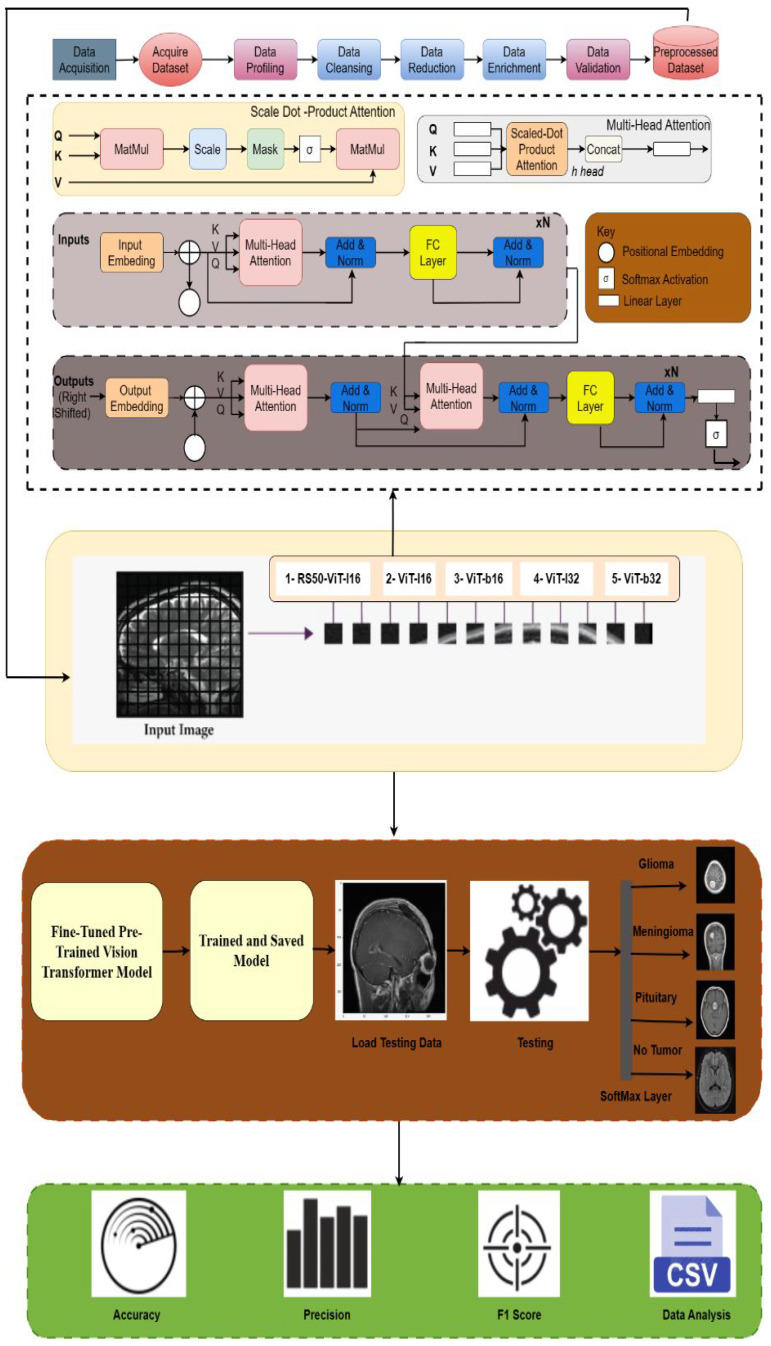
Process flow diagram of proposed work.

In this section, we harnessed the power of pre-trained vision transformer (ViT) models to advance the state of the art in medical image analysis, specifically for the classification of brain tumor images. We adopted a systematic approach by utilizing a set of pre-trained ViT models, namely R50-ViT-l16, ViT-l16, ViT-l32, ViT-b16, and ViT-b32, as the foundation for our work. Upon importing these pre-trained models, we meticulously tailored them to our task by implementing a custom classification architecture. Our approach involved constructing a neural network model using the TensorFlow framework, with the pre-trained ViT model as the initial layer. To adapt the models for accurate brain tumor classification, we introduced task-specific layers, including BatchNormalization and dense layers. These layers were essential for regularization and for enabling the model to recognize the intricate patterns inherent in medical images. Furthermore, we carefully fine-tuned hyperparameters, such as the learning rate and batch size, to ensure optimal model convergence and generalization. With a learning rate of 1 × 10 ^−4^ and the Adam optimizer, we conducted training for a total of 10 epochs on our extensive medical image dataset, continuously monitoring both training and validation performance. Graphical representation of the proposed model is shown in Figure 2.

#### 2.3.4. R50-ViT-l16 Model

The R50-ViT-l16 model is a hybrid model that combines the ResNet-50 convolutional neural network architecture with the ViT architecture. The ResNet-50 model extracts local features from the images, while the ViT-l16 model learns global features. The final classification is made by combining the local and global features. The ResNet-50 model has 50 layers, and it is made up of convolutional blocks and residual blocks. The convolutional blocks extract local features from the images, while the residual blocks help prevent the model from overfitting. Comprising 16 transformer layers, the ViT-L16 model is composed of both self-attention layers and feed-forward layers. The self-attention layers enable the model to concurrently focus on diverse regions of the image. Conversely, the feed-forward layers facilitate the acquisition of intricate image representations.

Table 2a shows the layer configuration of the R50-ViT-l16 model. The first layer, keras_layer, is a custom Keras layer that implements the ViT-L16 transformer architecture. The output of this layer is a 2048-dimensional vector. The next layer, flatten, flattens the 2048-dimensional vector into a 1-dimensional vector. This is performed to prepare the input for the next layer, batch_normalization. The batch_normalization layer normalizes the input vector. The next layer, dense, is a dense layer with 11 output units. This layer learns a classifier for the 11 classes of brain cancer. The batch_normalization layer normalizes the output of the dense layer. The final layer, dense, is a dense layer with four output units. This layer outputs the predicted class labels for the input image.

The total number of parameters in the R50-ViT-l16 model is 23,531,175. This includes 26,705 trainable parameters and 23,504,470 non-trainable parameters. The table also shows the output shape of each layer. The output shape of the first layer is (None, 2048), which means that the layer can output a batch of any size with 2048 features. The output shape of the final layer is (None, 4), which means that the layer can output a batch of any size with four features.

The R50-ViT-l16 model is a powerful model that can be used for brain tumor detection. It has a large number of parameters, which allows it to learn complex representations of images. However, it is also a computationally expensive model to train and deploy.

#### 2.3.5. ViT-b16 Model

The ViT-b16 model is a medium-scale ViT. It has 12 transformer encoder layers, each with 16 attention heads. This model underwent pre-training on the ImageNet dataset, which encompasses more than one million images. Subsequent to this pre-training phase, the model can be subjected to fine-tuning using a smaller dataset tailored to a specific image recognition task.

Table 2b shows the layer configuration of the ViT-b16 model. The first layer, ViT-b16, is a custom Keras layer that implements the ViT-B16 transformer architecture. The output of this layer is a 768-dimensional vector. The next layer, flatten, flattens the 768-dimensional vector into a 1-dimensional vector. This is performed to prepare the input for the next layer, batch_normalization. The batch_normalization layer normalizes the input vector. The next layer, dense, is a dense layer with 11 output units. This layer learns a classifier for the 11 classes of brain cancer. The batch_normalization layer normalizes the output of the dense layer. The final layer, dense, is a dense layer with four output units. This layer outputs the predicted class labels for the input image.

The total number of parameters in the ViT-b16 model is 85,810,279. This includes 85,808,721 trainable parameters and 1558 non-trainable parameters. The table also shows the output shape of each layer. The output shape of the first layer is (None, 768), which means that the layer can output a batch of any size with 768 features. The output shape of the final layer is (None, 4), which means that the layer can output a batch of any size with four features. The ViT-b16 model is a good choice for brain cancer detection when computational resources are limited. It has fewer parameters than the R50-ViT-L16 model, but it can still learn complex representations of images.

#### 2.3.6. ViT-l16 Model

The ViT-l16 model is a large-scale ViT. It has 16 transformer encoder layers, each with 32 attention heads. This model was pre-trained on the JFT-300M dataset containing over 300 million images. After pre-training, the model can be fine-tuned on a smaller dataset for a specific image recognition task.

Table 2c shows the layer configuration of the ViT-l16 model. The first layer, ViT-l16, is a custom Keras layer that implements the ViT-l16 transformer architecture. The output of this layer is a 1024-dimensional vector. The next layer, flatten, flattens the 1024-dimensional vector into a 1-dimensional vector. This is performed to prepare the input for the next layer, batch_normalization. The batch_normalization layer normalizes the input vector. The next dense layer is a dense layer with 11 output units. This layer learns a classifier for the 11 classes of brain cancer. The batch_normalization layer normalizes the output of the dense layer. The final layer, dense, is a dense layer with four output units. This layer outputs the predicted class labels for the input image.

The total number of parameters in the ViT-l16 model is 303,317,095. This includes 303,315,025 trainable parameters and 2070 non-trainable parameters. The table also shows the output shape of each layer. The output shape of the first layer is (None, 1024), which means that the layer can output a batch of any size with 1024 features. The output shape of the final layer is (None, 4), which means that the layer can output a batch of any size with four features.

The ViT-l16 model is a powerful model that can be used for brain cancer detection. It has a large number of parameters, which allows it to learn complex representations of images. However, it is also a computationally expensive model to train and deploy.

#### 2.3.7. ViT-l32 Model

The ViT-l32 model is a large-scale ViT. It has 24 transformer encoder layers, each with 32 attention heads. This model was pre-trained on the 21k-ImageNet dataset containing over 14 million images. After pre-training, the model can be fine-tuned on a smaller dataset for a specific image recognition task.

Table 2d shows the layer configuration of the ViT-l32 model. The first layer, ViT-l32, is a custom Keras layer that implements the ViT-l32 transformer architecture. The output of this layer is a 1024-dimensional vector. The next layer, flatten, flattens the 1024-dimensional vector into a 1-dimensional vector. This is performed to prepare the input for the next layer, batch_normalization. The batch_normalization layer normalizes the input vector. The next layer, dense, is a dense layer with 11 output units. This layer learns a classifier for the 11 classes of brain cancer. The batch_normalization layer normalizes the output of the dense layer. The final layer, dense, is a dense layer with four output units. This layer outputs the predicted class labels for the input image.

The total number of parameters in the ViT-l32 model is 305,525,863. This includes 305,523,793 trainable parameters and 2070 non-trainable parameters. The table also shows the output shape of each layer. The output shape of the first layer is (None, 1024), which means that the layer can output a batch of any size with 1024 features. The output shape of the final layer is (None, 4), which means that the layer can output a batch of any size with four features. The ViT-l32 model is the most powerful of the three models explained above. It can learn the most complex representations of images but is also the most computationally expensive to train and deploy.

#### 2.3.8. ViT-b32 Model

The ViT-b32 model is a specific implementation of the ViT architecture. It has 12 transformer encoder layers, each with 32 attention heads. This model was initially pre-trained using the ImageNet dataset, which includes a collection of over 1 million images. Following the pre-training phase, the model can then be fine-tuned on a more compact dataset targeted for a particular image recognition task. The model is also relatively efficient, requiring less computational resources to train than traditional convolutional neural networks (CNNs).

Table 2e shows the layer configuration of the ViT-b32 model. The first layer, ViT-b32, is a custom Keras layer that implements the ViT-b32 transformer architecture. The output of this layer is a 768-dimensional vector. The next layer, flatten, flattens the 768-dimensional vector into a 1-dimensional vector. This is performed to prepare the input for the next layer, batch_normalization. The batch_normalization layer normalizes the input vector. The next dense layer is a dense layer with 11 output units. This layer learns a classifier for the 11 classes of brain cancer. The batch_normalization layer normalizes the output of the dense layer. The final layer, dense, is a dense layer with four output units. This layer outputs the predicted class labels for the input image.

The total number of parameters in the ViT-b32 model is 87,466,855. This includes 87,465,297 trainable parameters and 1558 non-trainable parameters. The table also shows the output shape of each layer. The output shape of the first layer is (None, 768), which means that the layer can output a batch of any size with 768 features. The output shape of the final layer is (None, 4), which means that the layer can output a batch of any size with four features.

The ViT-b32 model is a good choice for brain cancer detection when computational resources are limited. It has fewer parameters than the ViT-l32 model, but it can still learn the complex representations of images.

## 3. Result

We used the following libraries for brain tumor image classification: NumPy, OpenCV, Scikit-learn, Keras, ViT-Keras, TensorFlow Hub, ImageDataGenerator from Keras, and pandas. We utilized the following metrics to evaluate our proposed model: accuracy and loss graphs, confusion matrix, and classification report.

- NumPy is a library developed for effectively managing large-scale, multi-dimensional arrays and matrices containing numerical data. Its significance extends to image processing and tasks within the domain of machine learning.
- OpenCV serves as a specialized library dedicated to image processing and computer vision. It encompasses a diverse array of functions enabling tasks like image reading, writing, display, and manipulation.
- Scikit-learn emerges as a machine-learning library equipped with a variety of algorithms tailor-made for tasks encompassing image classification.
- Keras operates as a high-level neural network API meticulously crafted for Python and constructed atop the TensorFlow framework. It simplifies the creation and training of neural networks, particularly those designed for image classification purposes.
- ViT-keras is a library for using ViT models for image classification in Keras. ViT models are a type of neural network designed to be more efficient than CNNs for image classification.
- TensorFlow Hub is a library for using pre-trained TensorFlow models in Keras. It makes using pre-trained models for image classification easy without retraining them.
- ImageDataGenerator from Keras is a library that can perform data augmentation on images. This helps to improve the performance of models by preventing overfitting.
- panda is a library for data analysis in Python. It makes it easy to load, manipulate, and analyze data in a tabular format.
- sklearn.model_selection is a library for performing model selection in Python. It can be used to split data into a training set and a test set and evaluate models’ performances.

We conducted our experiments on the Kaggle platform, utilizing the computational resources provided by Kaggle. Here are the specific computational resources we had access to: **Disk Space:** We were allocated 73.1 GB of disk space on Kaggle, which allowed us to store and manage the dataset, model checkpoints, and experimental results efficiently. **RAM (Random Access Memory):** Our Kaggle environment provided 13 GB of RAM. This was essential for loading and manipulating data, especially when working with large datasets and complex models. **GPU (Graphics Processing Unit):** We had access to a GPU with 15.9 GB of GPU memory. This GPU acceleration was crucial for training deep learning models, including the ViT models, efficiently and in a reasonable time frame. **Output Space:** Kaggle allocated 19.5 GB for output storage, enabling us to save and analyze model outputs, visualizations, and other experiment-related data.

We comprehensively overview various ViT models, key architectural details, and performance metrics. Table 3 summarizes the characteristics and performances of these models. Each row in the table corresponds to a specific ViT model, providing information about its patch size, backbone architecture, hidden units dimension, and ImageNet accuracy. For example, the R50-ViT-l16 model has a patch size 16 × 16, uses a ResNet-50 backbone, has 2048 hidden units, and achieves an ImageNet accuracy of 92.50%. ViT models with larger patch sizes, more hidden units, and stronger backbones tend to have better ImageNet accuracy. However, these models are also more computationally expensive to train and deploy. The R50-ViT-l16 model is a good compromise between performance and computational cost. It has a similar patch size and hidden units to the ViT-l16 model, but it uses the ResNet-50 backbone, allowing it to learn from the features that the ResNet-50 backbone has already learned. This leads to better performance on ImageNet without significantly increasing computational cost. Overall, the table provides a good overview of the available ViT models.

This result section explains the overall results of all applied models. The section consists of classification statistical values, a confusion matrix, and a graphical explanation of training and validation accuracy based on different parameters.

### 3.1. Training and Validation Accuracy and Loss

Both accuracy and loss are important metrics for assessing a model’s performance. Accuracy is a measure of how well the model predicts the correct output for a given input. It is calculated as the percentage of predictions that are correct. The concept of loss revolves around quantifying the disparity between a model’s predictions and the actual desired output. This measurement emerges as the summation of errors that the model incurs across all input instances. Additionally, training accuracy, training loss, and validation loss serve as gauges to evaluate a model’s performance in both the training and validation on ImageNet as shown in Table 4.

Elevated levels of training accuracy and validation accuracy signify a proficient model. However, an imbalance where training accuracy greatly surpasses the validation accuracy signifies potential overfitting. Overfitting emerges when the model becomes overly attuned to the intricacies of the training data, hindering its ability to generalize to novel data. To counter this, one can consider diminishing the model’s complexity or expanding the training dataset. Conversely, low values for both training and validation accuracy indicate underfitting. Underfitting transpires when the model lacks the intricacy needed to capture the underlying patterns within the data. In such instances, enhancing the model’s complexity or adopting an ensemble strategy can be beneficial.

**Figure 3:**
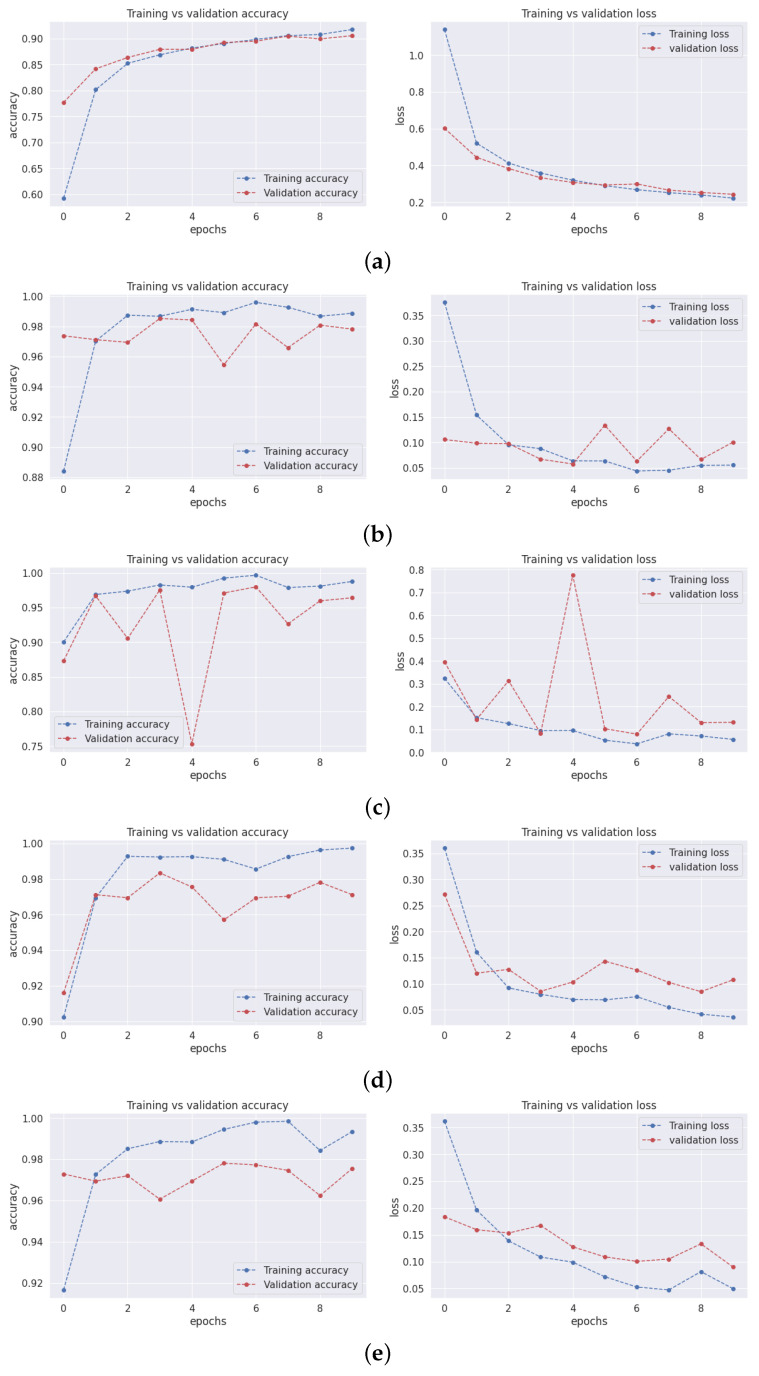
Training and loss graphs.

In Figure 3, the graphs present training and validation accuracy on the right axis, while the left axis showcases training and validation loss. These graphs span ten epochs, with the blue line depicting training values and the red line denoting validation values.

Observing the graphs, it is evident that the training accuracy across all models escalates with the progression of epochs. This is due to the models refining their capability to predict training data labels with greater precision. However, an interesting trend emerges where validation accuracy sometimes rises with increasing epochs. This could signify a form of overfitting, where models become excessively tailored to the training data. As a result, they are unable to generalize new, unforeseen data.

The training loss for all models decreases as the number of epochs increases. This is because the models improve their ability to fit the training data. However, the validation loss for the models sometimes decreases as the number of epochs increases. This is also a sign of overfitting.

The graphs for models 1 and 4 show that the validation accuracy and loss curves are relatively smooth. This suggests that the models are converging and generalizing well to new data. The graphs for models 2, 3, and 5 show that the validation accuracy and loss curves are more erratic. This suggests that the models may need to converge or generalize well to new data.

Overall, the results suggest that model 4 performs the best. The model can learn from the training data and generalize well to new data. Model 1 also performs well, but it may be starting to overfit the training data after a certain number of epochs. Models 2, 3, and 5 are not performing as well as models 1 and 4. They may need to be more balanced with the training data or generalize to new data.

### 3.2. Confusion Matrix

A confusion matrix proves to be a crucial tool for evaluating the efficacy of a classification model. It manifests as a tabular representation that offers insights into the model’s predictions. The table systematically displays the count of both accurate and erroneous predictions that the model generates for each individual class. Divided into four distinct quadrants, the confusion matrix categorizes diverse combinations of predicted and actual class labels. Within these quadrants, numerical values signify the instances falling within each category. The four quadrants are defined as follows:

- **True Positive (TePe)**: This quadrant accounts for instances where the model accurately predicts the positive class.
- **True Negative (TeNe)**: In this quadrant, the model precisely forecasts the negative class.
- **False Positive (FePe)**: This quadrant pertains to situations where the model predicts the positive class, although the actual class is negative.
- **False Negative (FeNe)**: This quadrant arises when the model forecasts the negative class, but the genuine class is positive.

The rows of the matrix depict the true tumor types, while the columns indicate the predicted tumor types. The numerical entries within the matrix cells provide a count of correctly and incorrectly classified samples.

**Figure 4:**
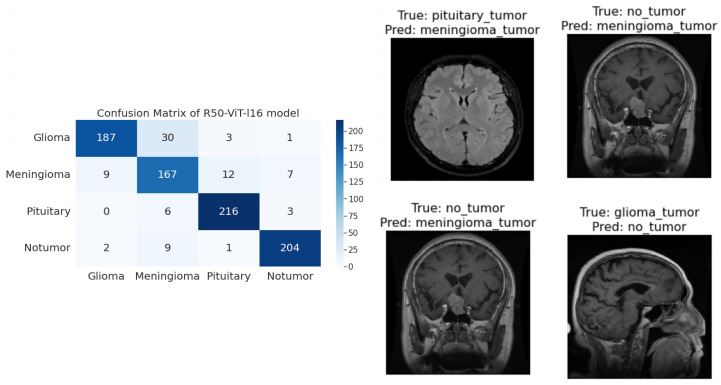
The right side of the figure shows a confusion matrix for model 1, and the left side of the figure shows images that were incorrectly classified by model 1.

**Figure 5:**
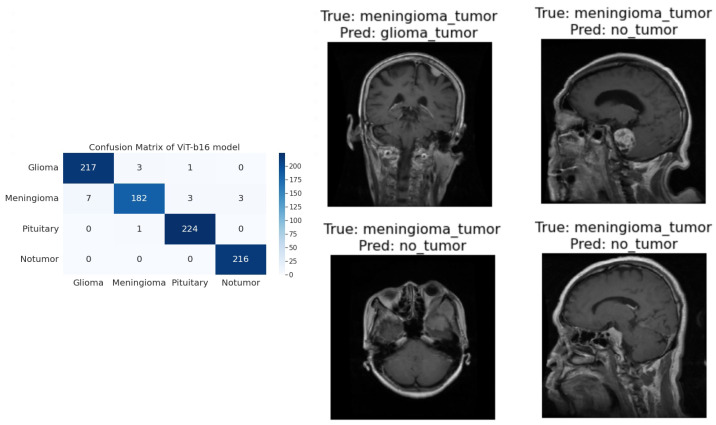
The right side of the figure shows a confusion matrix for model 2, and the left side of the figure shows images that were incorrectly classified by model 2.

**Figure 6:**
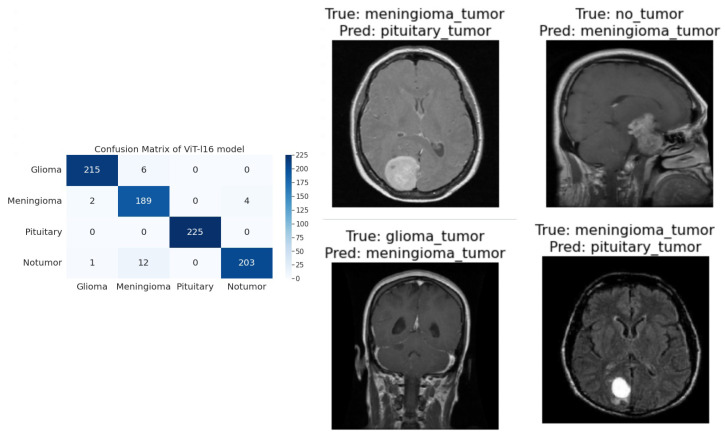
The right side of the figure shows a confusion matrix for model 3, and the left side of the figure shows images that were incorrectly classified by model 3.

**Figure 7:**
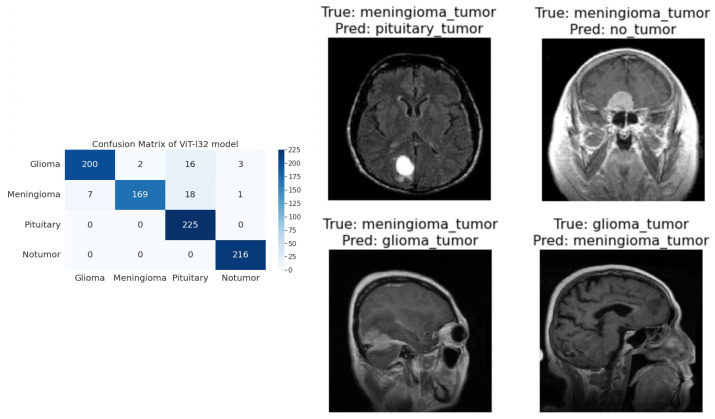
The right side of the figure shows a confusion matrix for model 4, and the left side of the figure shows images that were incorrectly classified by model 4.

**Figure 8:**
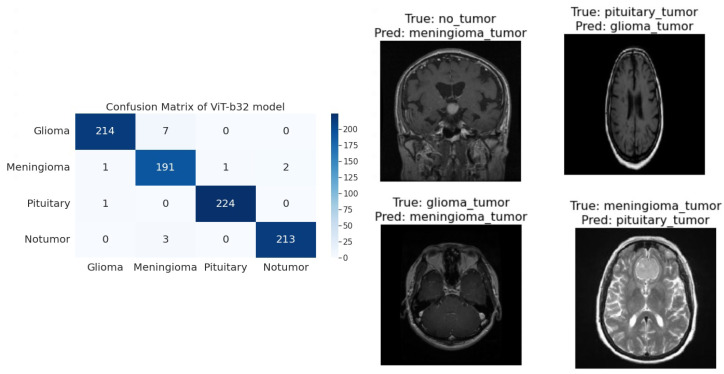
The right side of the figure shows a confusion matrix for model 5, and the left side of the figure shows images that were incorrectly classified by model 5.

Employing the confusion matrix, one can holistically assess a machine learning model’s performance through various metrics, such as:

- **R50-ViT-l16**
  − For Glioma tumor, the model correctly classified 187 images with an accuracy of 85.25%. The model also misclassified 30 Glioma images as Meningioma, 3 Glioma images as Pituitary, and 1 Glioma image as No Tumor.
  − For Meningioma tumor, the model correctly classified 167 Meningioma images, which is an accuracy of 85.58%. The model also misclassified 9 Meningioma images as Glioma, 12 Meningioma images as Pituitary, and 7 Meningioma images as No Tumor.
  − For Pituitary tumors, the model correctly classified 216 Pituitary images, with an accuracy of 96%. The model also misclassified six Pituitary images as Glioma, three Pituitary images as Meningioma, and three Pituitary images as No Tumor.
  − For the No Tumor class, the model correctly classified 204 No Tumor images, with an accuracy of 94.74%. The model also misclassified two Tumor images as Glioma, nine No Tumor images as Meningioma, and one No Tumor image as Pituitary.
  − Overall, the model has an accuracy of 90.31%. Its confusion matrix and incorrect classification images are shown in Figure 4. The model performs the best for Pituitary, with an accuracy of 96%. The model performs the worst for Glioma, with an accuracy of 85.25%.
- **ViT-b16**
  − For the Glioma tumor, the model correctly classified 217 images, with an accuracy of 98.19%. The model also misclassified three Glioma images as Meningioma, one Glioma image as Pituitary, and one Glioma images as No Tumor.
  − For Meningioma tumor, the model correctly classified 182 Meningioma images, with an accuracy of 93.33%. The model also misclassified seven Meningioma images as Glioma, three Meningioma images as Pituitary, and three Meningioma images as No Tumor.
  − For Pituitary tumors, the model correctly classified 224 Pituitary images, with an accuracy of 99.56%. The model also misclassified one Pituitary image as Glioma, zero Pituitary images as Meningioma, and zero Pituitary images as No Tumor.
  − For the No Tumor class, the model correctly classified 216 No Tumor images, which is an accuracy of 100%. The model also misclassified zero No Tumor images as Glioma, zero No Tumor images as Meningioma, and zero No Tumor images as Pituitary.
  − Overall, the model has an accuracy of 97.89%. Its confusion matrix and incorrect classification images are shown in Figure 5. The model performs best for No Tumor, with an accuracy of 100%. The model performs worst for Meningioma, with an accuracy of 93.33%.
- **ViT-l16**
  − For Glioma tumor, the model correctly classified 215 images as Glioma, with an accuracy of 97.73%. The model misclassified six Glioma images as Meningioma, zero Glioma images as Pituitary, and zero Glioma images as No Tumor.
  − For Meningioma tumor, the model correctly classified 189 images as Meningioma, achieving an accuracy of 94.50%. The model misclassified two Meningioma images as Glioma, zero Meningioma images as Pituitary, and four Meningioma images as No Tumor.
  − For Pituitary tumor, the model correctly classified 225 images as Pituitary, resulting in a perfect accuracy of 100%. The model did not misclassify any Pituitary images as other classes.
  − For the No Tumor class, the model correctly classified 203 images as No Tumor, with an accuracy of 94.88%. The model misclassified 1 Tumor image as Glioma and 12 Tumor images as Meningioma.
  − Overall, the model achieved an accuracy of approximately 97.08%. Its confusion matrix and incorrect classification images are shown in Figure 6. It performed best for the Pituitary tumor with a perfect accuracy of 100%, while the lowest accuracy was observed for the Meningioma tumor with an accuracy of 94.50%.
- **ViT-l32**
  − For Glioma tumor, the model correctly classified 200 images as Glioma, with an accuracy of 89.60%. The model misclassified 2 Glioma images as Meningioma, 16 Glioma images as Pituitary, and 3 Glioma images as No Tumor.
  − For Meningioma tumor, the model correctly classified 169 images as Meningioma, achieving an accuracy of 89.47%. The model misclassified 7 Meningioma images as Glioma, 18 Meningioma images as Pituitary, and 1 Meningioma image as No Tumor.
  − For Pituitary tumor, the model correctly classified 225 images as Pituitary, resulting in a perfect accuracy of 100%. The model did not misclassify any Pituitary images as other classes.
  − For No Tumor class, The model correctly classified 216 images as No Tumor, with an accuracy of 100%. The model did not misclassify any No Tumor images as other classes.
  − Overall, the model achieved an accuracy of approximately 94.51%. Its confusion matrix and incorrect classification images are shown in Figure 7. It performed the best for Pituitary and No Tumor, with perfect accuracies of 100%, while the lowest accuracy was observed for Glioma with an accuracy of 89.47%.
- **ViT-B32**
  − For Glioma tumor, the model correctly classified 214 images as Glioma, an accuracy of 96.39%. The model misclassified seven Glioma images as Meningioma, zero Glioma images as Pituitary, and zero Glioma images as No Tumor.
  − For Meningioma tumor, the model correctly classified 191 images as Meningioma, achieving an accuracy of 96.43%. The model misclassified one Meningioma image as Glioma, one Meningioma image as Pituitary, and two Meningioma images as No Tumor.
  − For Pituitary tumor, the model correctly classified 224 images as Pituitary, resulting in a perfect accuracy of 100%. The model misclassified one Pituitary image as Glioma, zero Pituitary image as Meningioma, and zero Pituitary images as No Tumor.
  − For the No Tumor class, the model correctly classified 213 images as No Tumor, with an accuracy of 98.61%. The model misclassified three Tumor images as Glioma, zero No Tumor images as Meningioma, and zero No Tumor images as Pituitary.
  − Overall, the model achieved an accuracy of approximately 98.24%. Its confusion matrix and incorrect classification images are shown in Figure 8. it performed the best for Pituitary with perfect accuracies of 100%, while the lowest accuracy was observed for Glioma with an accuracy of 96.39%.

### 3.3. Statistical Values of All Classes

A classification report serves as a metric for evaluating the performance of a trained classification model. This report presents precision (Pn), recall (Rl), F1 score (Fe), and support (St) metrics.

- **Precision (Pn)** Pn signifies the fraction of predicted positive instances that are truly positive.
  (1) $$\mathrm{Pn}=\frac{\mathrm{TePe}}{\mathrm{TePe}+\mathrm{FePe}}$$
- **Recall (Rl)** Rl denotes the fraction of actual positive instances that are correctly predicted as positive.
  (2) $$\mathrm{Rl}=\frac{\mathrm{TePe}}{\mathrm{TePe}+\mathrm{FeNe}}$$
- **F1 Score (Fe)** The Fe is a harmonic mean of Pn and Rl, providing a measure of the model’s accuracy. A perfect Fe is 1.0, while a score of 0.0 is undesirable.
  (3) $$\mathrm{Fe}=2\cdot \frac{\mathrm{Pn}\cdot \mathrm{Rl}}{\mathrm{Pn}+\mathrm{Rl}}$$
- **Support (St)** St corresponds to the number of instances in a class.
  (4) St=TePe+FePe+FeNe+TeNe

In this narrative comparison, we explore the performance metrics for each model across different tumor categories as shown in Table 5. The R50-ViT-l16 model demonstrated an accuracy of 90.31% overall. It achieved a Pn of 0.94 for the Glioma Tumor classification, while its Rl and Fe were 0.85 and 0.89, respectively. For Meningioma Tumor, the Pn was 0.79, the Rl was 0.86, and the Fe was 0.82. In contrast, the ViT-b16 model showcased remarkable accuracy at 97.89%. Its Pn for the Glioma Tumor classification was 0.97, with a Rl of 0.98 and an Fe of 0.98. The ViT-b16 model excelled in the Meningioma Tumor classification with a Pn of 0.98 while achieving a 0.93 Rl and 0.96 Fe. Overall, the ViT-b16 model demonstrated consistently high performance. The ViT_l16 model displayed commendable accuracy of 97.08%. It achieved a Pn of 0.99 for Glioma Tumors, a Rl of 0.97, and an Fe of 0.98. The model also showcased balanced performance for Meningioma Tumors, achieving a Pn of 0.91, Rl of 0.97, and Fe of 0.94. The ViT-l32 model exhibited an accuracy of 94.51%, with Pn, Rl, and Fes for Glioma Tumor at 0.97, 0.90, and 0.93, respectively. It excelled in the Pituitary Tumor classification with a perfect Rl of 1.00 and an Fe of 0.99. Lastly, the ViT-b32 model demonstrated the highest overall accuracy at 98.24%. It achieved a Pn of 0.99 for Glioma Tumors, a Rl of 0.97, and an Fe of 0.98. For Meningioma Tumor, the ViT-b32 model achieved a Rl of 0.98, and an impressive Fe of 0.96. Across these models, the ViT-b32 consistently displayed high performance, particularly excelling in No Tumor classification with the perfect Pn and Rl.

### 3.4. Comparison with Existing Model

In this section, we present a comprehensive comparison of our proposed approach against various existing state-of-the-art techniques for classification. Table 6 provides an overview of different classification methods, their corresponding accuracy rates, and the accuracy rates achieved by our proposed approach.

The referenced techniques encompass a range of methodologies, including complex network-based (CCN) classification, the utilization of GIST descriptors and extreme learning machines (ELM), attention modules combined with hypercolumn techniques and residual blocks, an improved version of ResNet, generative adversarial networks (GANs), and specific network architectures like BW-VGG-19, FT-Vit, and PatchResNet.

Comparatively, our proposed approach employs a variety of ViT models, each configured differently in terms of resolution and architecture. The resulting accuracy rates for our approach vary across these configurations. Notably, the R50-ViT-l16 variant yields an accuracy of 90.31%. Meanwhile, the ViT-b16 variant achieves an accuracy of 97.89%, closely followed by ViT-l16 with an accuracy of 97.08%. Similarly, ViT-l32 demonstrates an accuracy of 94.51%, and the ViT-b32 variant records the highest accuracy in our proposed approach at 98.24%.

Overall, our approach achieves competitive accuracy rates when compared to other methods. It can also achieve accurate results across various model configurations, making it suitable for a wide range of applications.

## 4. Discussion

The results section of this study presents a comprehensive analysis of the performance of various machine learning models in classifying different types of tumors in medical images. The models demonstrated strong performance across multiple tumor categories, with accuracy, Pn, Rl, and F1-score values ranging from 90.31% to 98.24%. The high accuracy levels indicate that the models could accurately predict tumor types. Precision values were consistently above 0.9 for each class, meaning the models could correctly identify positive cases with high confidence. Recall values were also high, exceeding 0.9, indicating that the models could capture true positive instances with high accuracy. F1 scores, representing a harmonic mean of precision and recall, were also consistently high, highlighting the models’ balanced performance.

The confusion matrices provided a detailed breakdown of each model’s performance, revealing where certain tumor types might pose challenges. However, despite these variations, all models exhibited an ability to classify tumor types with a high degree of accuracy. The graphs depicting training, validation accuracy, and loss offered insights into the models’ learning process. Early stopping is crucial to prevent overfitting, as validation accuracy plateaus or decreases after a certain number of epochs.

In this section, we compare and analyze the performance of the five ViT models based on the results presented in the previous section. The following subsections provide insights into the strengths and weaknesses of each model and highlight key observations regarding their classification capabilities. Comparing the overall accuracy and precision of the models, it is evident that Model 4 (ViT l32) achieved the highest accuracy of 94.51%, closely followed by Model 5 (ViT-B32) with an accuracy of 98.24%. Both models demonstrated exceptional precision scores across all tumor types, indicating their proficiency in correctly classifying instances within each category. While Model 5 exhibited outstanding precision and accuracy, it is noteworthy that Model 1 (R50-ViT-l16) demonstrated a higher recall rate for glioma tumors (85.71%) than Model 5’s recall rate of 97% for the same category. Model 3 (ViT l16) also demonstrated a balanced performance with recall rates above 90% for all tumor types. This suggests that Model 1 and Model 3 have a higher ability to identify instances of certain tumor types.

Analyzing the training and validation accuracy graphs, it becomes apparent that all models improved the training accuracy as the epochs increased. However, Models 2 (ViT b16) and 3 (ViT l16) demonstrated a decline in validation accuracy after several epochs, indicating potential overfitting. Model 5 (ViT-B32) showcased consistent validation accuracy, suggesting effective generalization to new data. The confusion matrices provide valuable insights into each model’s performance at a class level. Model 4 (ViT l32) demonstrated high accuracy and precision across all tumor categories, while Model 1 (R50-ViT-l16) exhibited a slightly lower precision for meningioma tumors. Model 5 (ViT-B32) balanced precision, recall, and F1-score, especially for pituitary tumors.

Model 2 (ViT b16) excelled in its precision for glioma tumors and overall accuracy, showcasing its capability to classify glioma instances with high confidence. Model 4 (ViT l32) stood out for its consistent performance across all metrics, making it a robust candidate for tumor classification. Model 3 (ViT l16) demonstrated a balanced performance with commendable recall rates, while Model 1 (R50-ViT-l16) exhibited competitive accuracy and recall for different tumor types.

Comparing the models, ViT-B32 (Model 5) had the highest overall accuracy of 98.24%. This suggests that more complex architectures can contribute to improved performance. While the study’s findings are promising, there are some limitations. The generalization of diverse medical images and cases needs further exploration. Additionally, considering sensitivity, specificity, and practical deployment aspects could enhance the models’ real-world applicability. This research underscores the potential of machine learning models, particularly ViT-based architectures, in accurately classifying tumor types in medical images. The robust accuracy, precision, and recall scores validate their utility in medical diagnosis. However, addressing overfitting and considering practical implementation remains vital. This study provides a foundation for future medical image analysis and diagnosis advancements.

## 5. Conclusions

The ViT models demonstrated strong performance in classifying different types of tumors in medical images. The models achieved high accuracy, precision, recall, and F1-score values, ranging from 90.31% to 98.24%. The confusion matrices provided a detailed breakdown of each model’s performance, revealing where certain tumor types might pose challenges. ViT-b32 had the highest overall accuracy of 98.24%, followed by ViT-l32 with an accuracy of 94.51%. ViT-b32 also demonstrated the best precision and recall scores across all tumor types. R50-ViT-l16 exhibited a higher recall rate for glioma tumors than ViT-b32, but a slightly lower precision for meningioma tumors. ViT-b16 excelled in its precision for glioma tumors and overall accuracy, while ViT-l16 demonstrated a balanced performance with commendable recall rates. The study’s findings suggest that more complex architectures can contribute to improved performance in tumor classification. While the study’s findings are promising, there are some limitations. The generalization of diverse medical images and cases needs further exploration. Additionally, considering sensitivity, specificity, and practical deployment aspects could enhance the models’ real-world applicability. This research underscores the potential of ViT models, in accurately classifying tumor types in medical images. The robust accuracy, precision, and recall scores validate their utility in medical diagnosis. However, addressing overfitting and considering practical implementation remains vital. This study provides a foundation for future medical image analysis and diagnosis advancements. Overall, the results of this study are promising and suggest that ViT models have the potential to revolutionize tumor classification in medical imaging. However, further research is needed to address the limitations of this study and to ensure that these models can be effectively deployed in real-world settings.

## Figures and Tables

**Table 1:** Brain tumor image dataset statistics.

Class Name	Number of Images	Training	Testing
Glioma	1321	1100	221
Meningioma	1339	1144	195
No tumor	1595	1241	216
Pituitary	1457	1370	225
Total	5712	4855	857

**Table 2:** Models using ViT.

(a) Model Vision_Transformer R50-vit-l16		
**Layer (Type)**	**Output Shape**	**Parameters**
keras_layer (KerasLayer)	(None, 2048)	23,500,352
Flatten	(None, 2048)	0
batch_normalization	(None, 2048)	8192
Dense	(None, 11)	22,539
batch_normalizatio	(None, 11)	44
Dense	(None, 4)	48
**(b) Model Vision_Transformer vit_b16**		
**Layer (Type)**	**Output Shape**	**Parameters**
vit-b16 (Functional)	(None, 768)	85,798,656
Flatten	(None, 768)	0
batch_normalization	(None, 768)	3072
Dense	(None, 11)	8459
batch_normalization	(None, 11)	44
Dense	(None, 4)	48
**(c) Model Vision_Transformer vit_l16**		
**Layer (Type)**	**Output Shape**	**Parameters**
vit-l16 (Functional)	(None, 1024)	303,301,632
Flatten	(None, 1024)	0
batch_normalization	(None, 1024)	4096
Dense	(None, 11)	11,275
batch_normalization	(None, 11)	44
Dense	(None, 4)	48
**(d) Model Vision_Transformer vit_l32**		
**Layer (Type)**	**Output Shape**	**Parameters**
vit-l32 (Functional)	(None, 1024)	305,510,400
Flatten	(None, 1024)	0
batch_normalization	(None, 1024)	4096
Dense	(None, 11)	11,275
batch_normalization	(None, 11)	44
Dense	(None, 4)	48
**(e) Model Vision_Transformer vit_b32**		
**Layer (Type)**	**Output Shape**	**Parameters**
vit-b32 (Functional)	(None, 768)	87,455,232
Flatten	(None, 768)	0
batch_normalization	(None, 768)	3072
Dense	(None, 11)	8459
batch_normalization	(None, 11)	44
Dense	(None, 4)	48

**Table 3:** Table of ViT models with their patch size, backbone, hidden units, and ImageNet accuracy.

Model	Patch Size	Backbone	Hidden Units	ImageNet Accuracy
R50-ViT-l16	16 × 16	ResNet-50	2048	92.50%
ViT-b16	16 × 16	ViT-B16	1024	90.20%
ViT-l16	16 × 16	ViT-L16	1280	92.30%
ViT-l32	16 × 16	ViT-L32	2560	93.50%
ViT-b32	32 × 32	ViT-B32	2048	91.10%

**Table 4:** Results of the models.

Model	Training Accuracy	Validation Accuracy	Training Loss	Validation Loss
R50-ViT-l16	0.98	0.90	0.03	0.05
ViT-b16	0.99	0.97	0.01	0.10
ViT-l16	0.98	0.97	0.08	0.15
ViT-l32	1.00	0.94	0.04	0.12
ViT-b32	0.98	0.98	0.04	0.12

**Table 5:** Model performance comparison for different tumor categories.

Tumor Category	R50-ViT-l16	ViT_b16	ViT_l16	ViT_l32	ViT_b32
	Pn: 0.94	Pn: 0.97	Pn: 0.99	Pn: 0.97	Pn: 0.99
Glioma Tumor	Rl: 0.85	Rl: 0.98	Rl: 0.97	Rl: 0.90	Rl: 0.97
	Fe: 0.89	Fe: 0.98	Fe: 0.98	Fe: 0.93	Fe: 0.98
	Pn: 0.79	Pn: 0.98	Pn: 0.91	Pn: 0.99	Pn: 0.95
Meningioma Tumor	Rl: 0.86	Rl: 0.93	Rl: 0.97	Rl: 0.87	Rl: 0.98
	Fe: 0.82	Fe: 0.96	Fe: 0.94	Fe: 0.92	Fe: 0.96
	Pn: 0.92	Pn: 0.98	Pn: 1.00	Pn: 0.86	Pn: 1.00
No Tumor	Rl: 0.94	Rl: 1.00	Rl: 0.94	Rl: 1.00	Rl: 0.99
	Fe: 0.93	Fe: 0.99	Fe: 0.97	Fe: 0.93	Fe: 0.99
	Pn: 0.96	Pn: 0.99	Pn: 0.98	Pn: 0.98	Pn: 0.99
Pituitary Tumor	Rl: 0.96	Rl: 1.00	Rl: 1.00	Rl: 1.00	Rl: 1.00
	Fe: 0.96	Fe: 0.99	Fe: 0.99	Fe: 0.99	Fe: 0.99
**Overall Accuracy**	**90.31%**	**97.89%**	**97.08%**	**94.51%**	**98.24%**

**Table 6:** Comparison with the existing state-of-the-art work.

No	Classification Method	Accuracy
[21]	CCN based on complex networks	95.49%
[22]	BW-VGG-19	98%
[23]	PatchResNet	98.10%
[24]	Improved ResNet	91.30%
[25]	GAN	96%
[26]	GIST descriptor and ELM	94.93%
[27]	FT-Vit	98.13%
[28]	Hypercolumn technique, and Residual block	97.69%
**Our Proposed Approach**	R50-ViT-l16	90.31%
	ViT-b16	97.89%
	ViT-l16	97.08%
	ViT-l32	94.51%
	ViT-b32	98.24%

## Data Availability

Dataset and code can be shared upon certain request to corresponding author.
